# Magnetocardiography in diagnosis of MINOCA: a case series

**DOI:** 10.3389/fcvm.2026.1759132

**Published:** 2026-07-15

**Authors:** Xiaoyan Xue, Jiandong Zhang, Yuheng Zhou, Yihao Zhu, Jiaqi Liang, Kangqi Tian, Dong Xu, Yuguo Chen, Xu Zhang, Min Xiang

**Affiliations:** 1 Key Laboratory of Ultra-Weak Magnetic Field Measurement Technology, Chinese Ministry of Education, School of Instrumentation and Optoelectronic Engineering, Beihang University, Beijing, China; 2 Hangzhou Innovation Institute, Beihang University, Hangzhou, China; 3 Shandong Key Laboratory: Magnetic Field-free Medicine & Functional Imaging, Jinan, China; 4 Research Institute of Shandong University: Magnetic Field-free Medicine & Functional Imaging, Jinan, China; 5 Senior Department of Urology, Chinese PLA General Hospital, Beijing, China; 6 National Institute of Extremely-weak Magnetic Field Infrastructure, Hangzhou, China; 7 Chest Pain Center, Qilu Hospital of Shandong University, Jinan, China; 8 Key Laboratory of Cardiovascular Remodeling and Function Research, Chinese Ministry of Education, National Health Commission and Chinese Academy of Medical Sciences, Qilu Hospital of Shandong University, Jinan, China; 9 Department of Emergency Medicine, Qilu Hospital of Shandong University, Jinan, China; 10 Shandong Provincial Clinical Research Center for Emergency and Critical Care Medicine, Institute of Emergency and Critical Care Medicine of Shandong University, Chest Pain Center, Qilu Hospital of Shandong University, Jinan, China; 11 Hefei National Laboratory, Hefei, China

**Keywords:** case report, chest pain, MCG, MINOCA, SERF atomic magnetometer

## Abstract

**Background:**

Currently, there is a paucity of accurate, convenient, rapid, and efficient diagnostic approaches for myocardial infarction with non-obstructive coronary arteries (MINOCA) in patients who have undergone coronary angiography (CAG) or coronary computed tomography angiography (CCTA) confirming non-obstructive coronary arteries, particularly when the ECG is normal or non-diagnostic. Given that MINOCA is pathologically characterized by myocardial ischemia and abnormal local electrical activity, magnetocardiography (MCG), which can sensitively detect changes in myocardial depolarization and repolarization current density, may provide a promising diagnostic option.

**Case summary:**

This case series includes 2 patients with non-ST-segment elevation myocardial infarction (NSTEMI), both of whom were confirmed to have a subtype: MINOCA, and all underwent MCG. In both cases, serial ECG findings were normal, yet myocardial enzymes were elevated (suggestive of infarction); thus to resolve this diagnostic ambiguity, we assessed infarction-related waveform features by referencing previously observed infarction characteristics and confirmed that even without ECG-detectable infarction, MCG waveforms still exhibit highly prominent infarction-related features. Subsequent cardiac catheterization confirmed no coronary artery stenosis in both patients. In the second case, cardiac magnetic resonance (CMR) further completed the guideline-specified final diagnostic workflow for MINOCA, which retrospectively validated the diagnostic accuracy of MCG.

**Conclusion:**

In real-world clinical practice, conventional diagnostic pathways frequently face challenges in the underdiagnosis and suboptimal classification of MINOCA. As a rapid, painless, non-invasive, and radiation-free technique, MCG may serve as a complementary functional assessment tool for patients with suspected MINOCA and elevated myocardial biomarkers. Based on these preliminary case observations, MCG shows potential to identify subtle electrical abnormalities related to myocardial ischemia that are not readily captured by routine examinations. This study provides exploratory evidence supporting the possible additive value of MCG in refining the diagnostic workflow of MINOCA, and warrants further larger-scale investigations to validate its clinical utility, including differentiation from other non-ischemic myocardial injury conditions.

## Introduction

Even in the contemporary era, the integration of findings from multimodality diagnostic approaches for myocardial infarction with non-obstructive coronary arteries (MINOCA)—a condition estimated to have an overall prevalence of 6% among patients with acute myocardial infarction—is well endorsed in diagnostic guidelines, which recommend modalities such as cardiac magnetic resonance (CMR), optical coherence tomography (OCT), and intravascular ultrasound (IVUS) (1). However, MINOCA frequently remains a provisional diagnosis, with up to 43% of cases receiving inconclusive diagnoses due to practical barriers to invasive testing and challenges in etiological identification (2). Notably, extant research has devoted comparatively less attention to its diagnosis within real-world medical practice (3–7). Furthermore, a minority of patients with MINOCA display normal electrocardiographic or cardiac troponin levels on arrival—adding another layer of difficulty to clinical diagnosis. In such scenarios, the utility of novel diagnostic approaches may improve efficiency as well as diagnostic accuracy.

**Figure 1:**
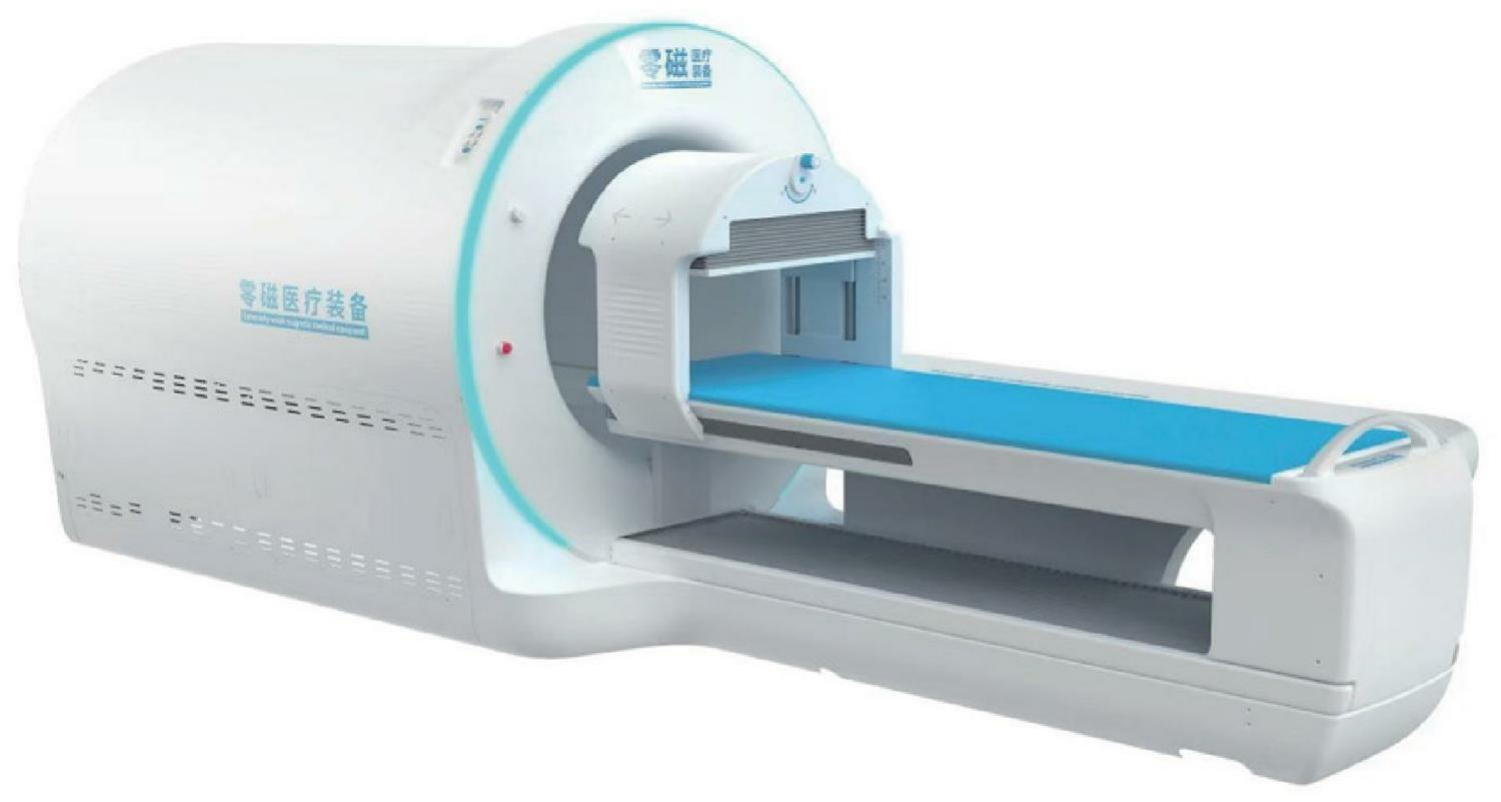
Image of the SERF-MCG magnetocardiograph system.

Magnetocardiography (MCG) has emerged as a promising non-invasive tool for detecting myocardial ischemia, yet to our knowledge, no prior studies have specifically evaluated its diagnostic utility in MINOCA. Notably, for patients with this condition, MCG may offer the potential to optimize its diagnostic workflow. This case series explores the clinical application of MCG, captured by the 36-channel spin exchange relaxation-free (SERF) magnetocardiography system (Hangzhou Lingci Medical Equipment, LMCG-36A), as a rapid diagnostic tool in patients with MINOCA presenting with normal electrocardiographic findings in the clinical setting (Figure 1).

**Figure 2:**
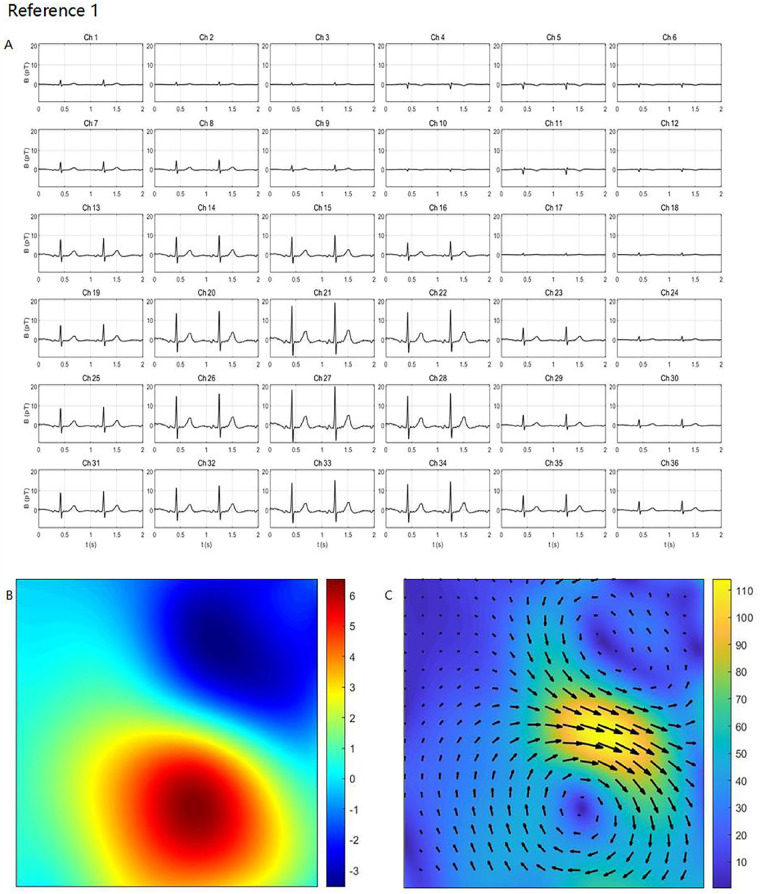
MCG waveform characteristics of myocardial ischemia and infarction. Refs. (1, 2) show normal MCG controls. Abnormal ischemic features in unstable angina (UA), non-ST-elevation myocardial infarction (NSTEMI), and ST-elevation myocardial infarction (STEMI) are marked with red arrows and boxes. Both magnetic field and current density maps were acquired at the *T*-wave peak.

Similar to the ECG waveform, time line MCG (TLMs) serves as a mainstay for the detection of myocardial infarction. Based on our prior analysis of TLMs features in myocardial infarction (MI), we observed that TLMs in MI cases show flattened or downsloping ST segments, flattened or inverted T waves and pathological *Q*—including deep *Q* waves, *Q*r waves, and fragmented *Q* waves—changes not observed in healthy individuals. Notably, pathological *Q* waves were absent in MCG recordings from patients with dilated cardiomyopathy, suggesting that this specific MCG feature may be uncommon in non-ischemic cardiomyopathies and thus potentially useful for distinguishing MI from these conditions (Supplementary Figure S1). Among these TLM alterations, the ST-segment and *T*-wave changes exist in both MI and ischemic patients, such as those with unstable angina (UA), and the pathological *Q* waves are specific to MI, as illustrated in Figure 2. Meanwhile, we conducted a comparative analysis of magnetic field maps and current density maps among MI patients, patients with UA, and healthy controls. We found that TLMs of patients with MINOCA exhibit distinct morphological differences compared with patients with isolated myocardial ischemia and healthy controls. Notably, these differences are further corroborated by the variations observed in two-dimensional magnetic field maps and current density maps. Collectively, these observations confirm that the aforementioned infarction-related characteristics of TLM can serve as easily identifiable indicators for distinguishing MINOCA patients from those with myocardial ischemia and healthy individuals (Figure 2). Therefore, we take the TLMs, magnetic field maps and current density maps from patients with MI, patients with UA, and healthy individuals as references. Based on this diagnostic framework, herein, we present MCG waveforms from 2 cases of MINOCA, further evaluate the abnormal morphological characteristics of these waveforms, and report them as part of a clinical study conducted at the cardiac outpatient clinic of a tertiary academic medical center. This report aims not only to share our center's diagnostic experience with magnetocardiography in ischemic heart disease, but also to evaluate the translational potential of applying classic ECG ischemic features to visual MCG analysis.

**Figure:**
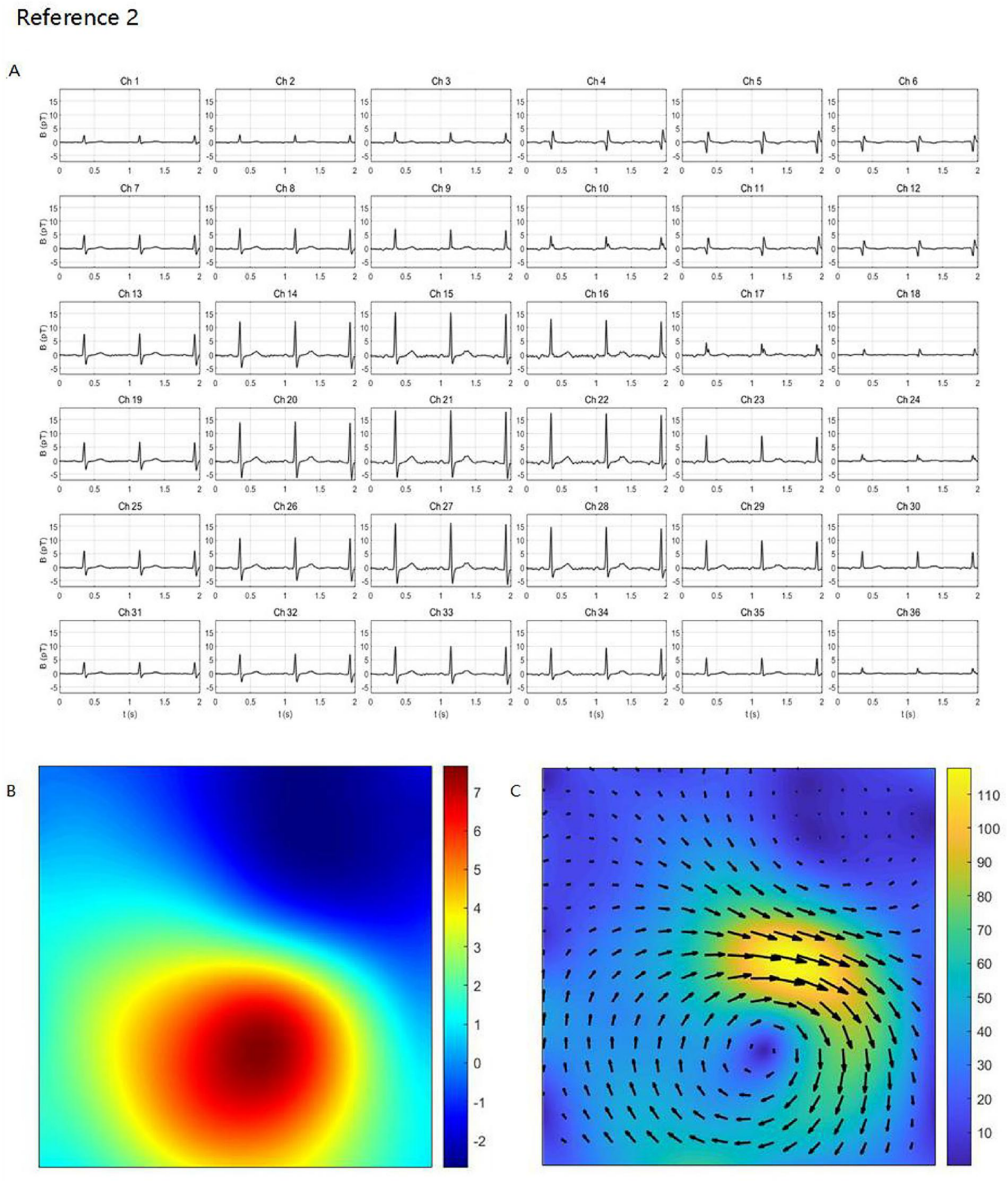


**Figure:**
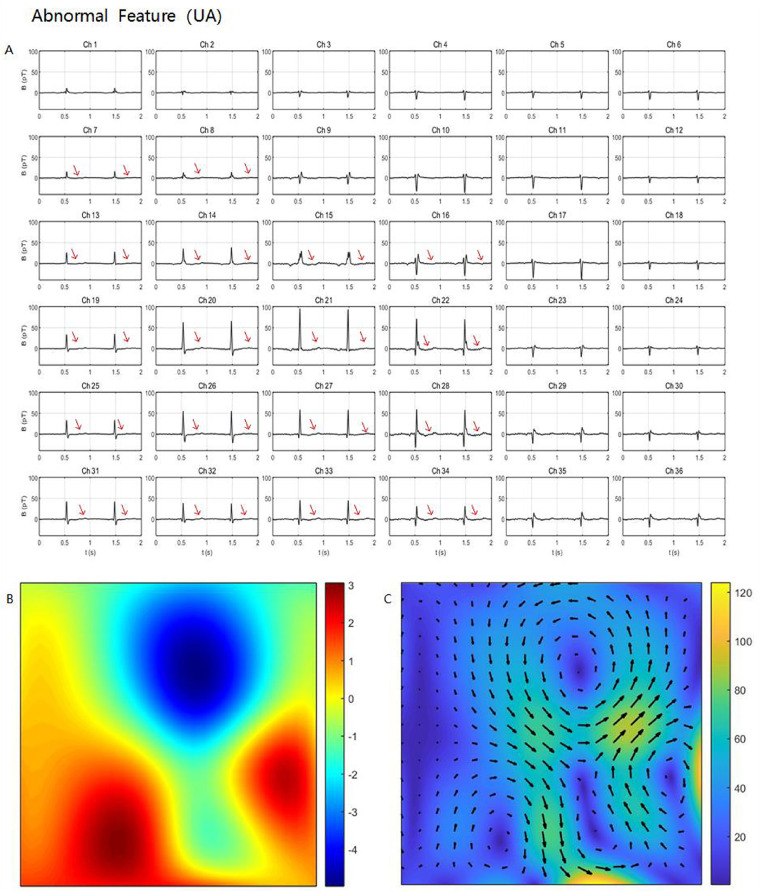


**Figure:**
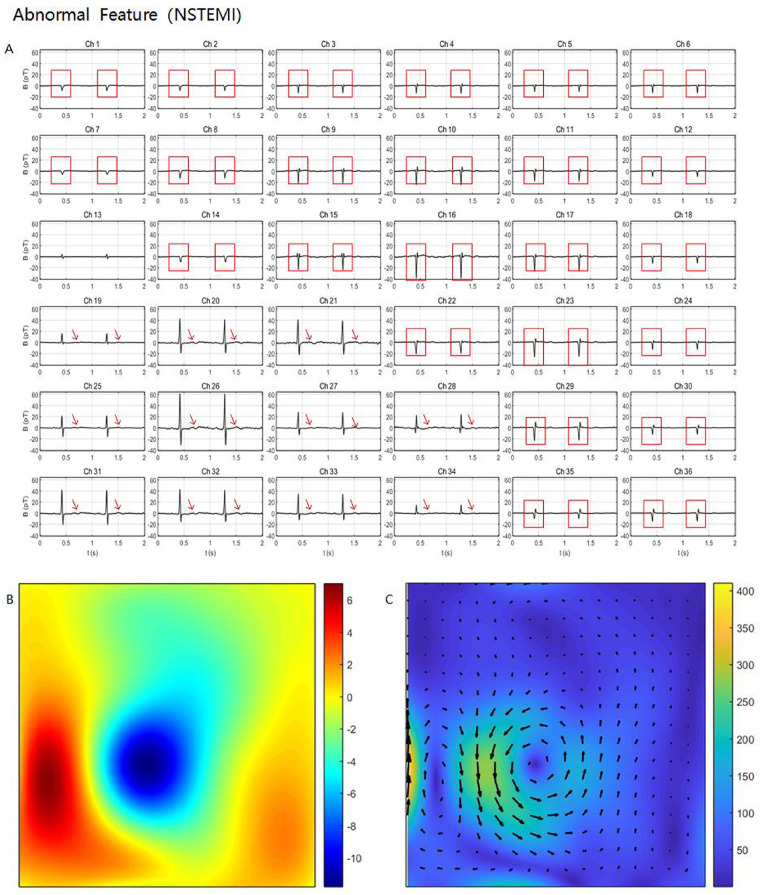


**Figure:**
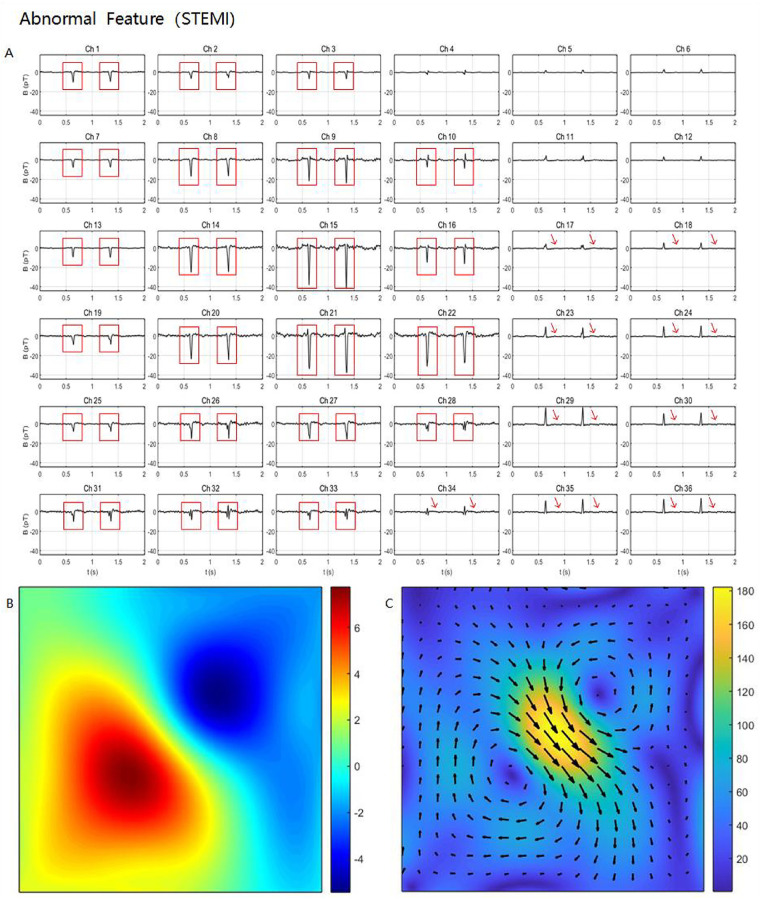


## Methods

### Data acquisition

A self-developed 36-channel SERF-MCG system to perform the MCG recordings (Figure 1). In brief, a patient lay in the supine position and the arrayed sensors on the acquisition panel positioned close to, but not in contact with chest wall. Data were then collected after positioning the laser at the top of the sternum. MCG recordings were carried out at rest for 180 s, at a sampling rate of 1,000 Hz, covering a bandwidth from DC to 100 Hz, and the operating dynamic range is ±20 nT. The raw MCG signals were initially recorded within a DC-100 Hz bandwidth without any real-time filtering. All patient recordings were made in the absence of magnetically shielded room.

### Electromagnetic shielding and signal processing

The electromagnetic shielding structure typically consists of 3–5 layers. The inner layer is made of high-permeability permalloy, which is designed to enhance the shielding performance against low-frequency magnetic fields. The middle layer is a metal layer of copper or aluminum, functioning to shield radio frequency signals and electric field interference. The outer layer is composed of a thick-walled steel plate or an additional *µ*-metal layer, aimed at increasing the shielding depth. Collectively, these layers work together to effectively shield both static and low-frequency magnetic fields, as well as RF and electric field disturbances.

Even within a heavily shielded environment, multiple noise contributors—including external electromagnetic leakage, internal device noise, and biological artifacts—can overlap and degrade signal quality. Consequently, no single filter can effectively eliminate all noise types, necessitating a multi-layered filtering strategy to achieve a high signal-to-noise ratio and satisfy the precision requirements of downstream analyses.

Given the extremely small amplitude of MCG signals (on the order of picoteslas), signal quality optimization during preprocessing was essential. A multi-layered filtering strategy was adopted: (1) A 50 Hz low-pass filter (4th-order Butterworth, −40 dB/octave) to suppress high-frequency interference; (2) A 1–45 Hz bandpass filter (4th-order Butterworth, −40 dB/octave) to retain the main physiological components while removing baseline drifts and high-frequency noise; (3) A 50 Hz notch filter (*Q* = 30, −60 dB) to eliminate powerline interference. All filters and averaging were applied during software-based post-acquisition preprocessing.

### MCG waveform interpretation and diagnostic criteria

- Waveform Analysis and Interpretation Procedure

MCG waveform analysis was performed using a novel ECG-like analytical approach, which was preliminarily validated in our large-scale machine learning study (*n* = 4,500 MCG recordings, under review). To ensure objective interpretation, two senior electrocardiographers, each having undergone extensive training on a dedicated database of more than 2,000 normal MCG tracings, conducted blinded and independent assessments. Any discrepancies between their initial interpretations were resolved through a consensus process involving a third senior expert.

- Establishment and Application of Normal Reference Ranges

Normal reference ranges for all key MCG parameters (e.g., R_amp, T_amp, ratio_RT, Q_amp, k1–k4_amp) were established from a large, rigorously defined healthy cohort, as detailed in the Supplementary Materials (“Establishment of Normal Reference Ranges”) and are summarized in Supplementary Table S1. These ranges define the central 95% distribution (2.5th–97.5th percentiles) for each parameter. The normal reference ranges (2.5th–97.5th percentiles) for three key waveform parameters—*Q*-wave amplitude (*Q*_amp), *R*/*T* ratio, and *T*-wave amplitude (*T*_amp)—across all 36 channels are detailed in Table 1. The reference ranges for additional parameters are detailed in the Supplementary File titled “Statistical Table of 36-Channel Parameter Data”.

- Quantitative Definition of Abnormalities

**Table 1:** Normal reference ranges for *Q*_amp, *R*/*T* ratio, and T_amp across 36 channels .

NO.	*Q*_amp (pT)	*R*/*T* ratio	*T*_amp (pT)	NO.	*Q*_amp (pT)	*R*/*T* Ratio	*T*_amp (pT)	NO.	*Q*_amp (pT)	*R*/*T* Ratio	*T*_amp (pT)
1	[0.492, 0.565]	[6.167, 6.817]	[0.523, 0.588]	2	[0.512, 0.596]	[5.613, 6.242]	[0.639, 0.706]	3	[0.601, 0.685]	[2.651, 2.887]	[1.508, 1.640]
4	[0.671, 0.768]	[1.849, 1.972]	[2.657, 2.862]	5	[1.941, 2.283]	[1.769, 1.886]	[2.512, 2.698]	6	[2.279, 2.635]	[1.741, 1.856]	[2.309, 2.500]
7	[0.422, 0.469]	[5.113, 5.651]	[0.906, 1.003]	8	[0.419, 0.473]	[5.788, 6.392]	[1.129, 1.251]	9	[0.473, 0.526]	[4.877, 5.20]	[1.880, 2.077]
10	[0.583, 0.650]	[2.232, 2.399]	[4.190, 4.560]	11	[0.624, 0.712]	[1.808, 1.928]	[3.964, 4.278]	12	[3.112, 3.557]	[1.832, 1.964]	[2.062, 2.220]
13	[0.663, 0.725]	[4.799, 5.205]	[1.791, 1.957]	14	[0.747, 0.835]	[5.329, 5.803]	[2.812, 3.076]	15	[0.766, 0.863]	[8.493, 9.361]	[2.817, 3.110]
16	[0.757, 0.863]	[6.551, 7.415]	[3.105, 3.460]	17	[2.978, 3.465]	[2.456, 2.685]	[3.090, 3.403]	18	[4.415, 4.911]	[2.591, 2.837]	[1.577, 1.727]
19	[0.858, 0.930]	[4.600, 4.934]	[2.351, 2.550]	20	[1.386, 1.518]	[4.780, 5.118]	[5.029, 5.437]	21	[2.362, 2.640]	[5.708, 6.126]	[7.195, 7.776]
22	[4.518, 5.091]	[8.012, 8.802]	[4.241, 4.642]	23	[5.559, 6.112]	[5.470, 6.128]	[1.662, 1.825]	24	[4.873, 5.264]	[5.113, 5.717]	[0.823, 0.910]
25	[0.823, 0.892]	[4.563, 4.894]	[2.119, 2.311]	26	[1.674, 1.813]	[4.513, 4.806]	[4.856, 5.246]	27	[3.157, 3.470]	[4.784, 5.096]	[7.514, 8.091]
28	[5.296, 5.843]	[5.268, 5.710]	[5.647, 6.104]	29	[5.384, 5.836]	[5.178, 5.731]	[2.126, 2.325]	30	[3.721, 3.996]	[6.624, 7.406]	[0.636, 0.703]
31	[1.085, 1.186]	[4.702, 5.054]	[2.658, 2.924]	32	[1.335, 1.449]	[4.863, 5.219]	[2.972, 3.224]	33	[2.304, 2.516]	[4.988, 5.338]	[4.328, 4.688]
34	[2.885, 3.164]	[5.358, 5.838]	[3.168, 3.448]	35	[3.239, 3.523]	[5.217, 5.762]	[1.553, 1.702]	36	[3.112, 3.385]	[5.545, 6.120]	[0.810, 0.904]

No., Channel number in the multichannel MCG system. Ranges: 2.5th–97.5th percentiles.

Abnormalities were defined as quantitative deviations from the established normal reference ranges. Specifically, for any given channel, a parameter value was classified as abnormal if it fell below the 2.5th percentile or above the 97.5th percentile. This objective threshold (corresponding to a 95% reference interval) forms the basis for all subsequent diagnostic classifications.

- Morphological Definition of Ischemia-Related Patterns

Building upon these quantitative thresholds, ischemia-related patterns were defined using specific ECG-equivalent morphological features, the validity of which was confirmed within our cohort. The operational definitions for these composite features, which integrate the abnormal parameter criteria, are provided in Table 2.

**Table 2:** Definitions and abnormality criteria for MCG morphological features.

Feature	Definition		Reference		Abnormal	
ST segment	the magnetic interval between the J point and the *T*-wave onset.	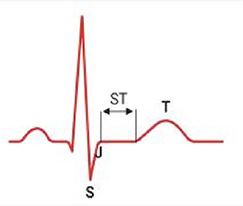	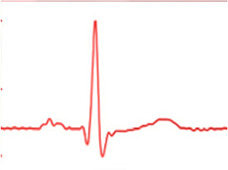	Mild upward-sloping ST elevation, with no significant horizontal or downsloping ST depression.	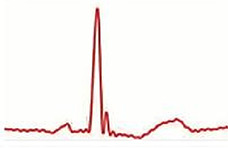	Significant depression relative to baseline, horizontal/downsloping depression, suggestive of ischemia.
T amplitude	the maximum magnetic field strength measured from the isoelectric baseline to the peak of the T wave in MCG.	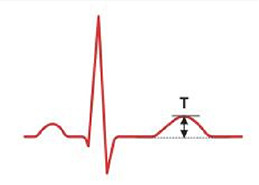	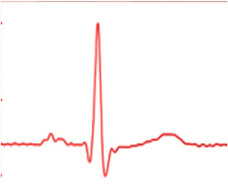	≥1/10 of the corresponding R-wave height in the same channel (in R-wave positive channels)	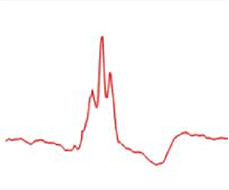	<1/10 of the corresponding R-wave height in the same channel, suggestive of myocardial ischemia. *T*-wave inversion or biphasic T waves (in R-wave positive channels).
Pathological Q wave	A pathological Q wave in MCG is a negative magnetic deflection indicative of myocardial necrosis or fibrosis, defined by two criteria.	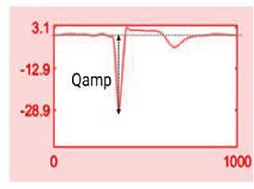	Q waves were present only in channels 5, 6, 12, 17, 18, 21–24, 27–30, and 35–36, with amplitudes within the nominal reference ranges (generally < 6 pT). In all other channels, Q waves were absent or of negligible amplitude.		Either: (1) The presence of a Q wave in a non-physiological channel, or (2) A Q wave in a physiological channel with a magnetic amplitude exceeding 10 pT (the reference range for the corresponding channel).	

## Case presentation

### Case 1

A 53-year-old woman presented to the emergency department with chest pain as the chief complaint. The pain was retrosternal in location, squeezing and pressing in character, with radiation to the back, lasting for approximately 1 day. She reported recurrent episodes of chest pain over the past two months. The patient had a history of grade 3 hypertension, managed with diltiazem (a calcium channel blocker) and telmisartan (an angiotensin II receptor blocker). The patient denied a history of diabetes, hyperlipidemia, smoking, or alcohol use. Vital signs on admission were as follows: temperature 36.4 °C, heart rate 80 beats per min (bpm), respiratory rate 18 bpm, and blood pressure 116/74 mmHg.

**Figure 3:**
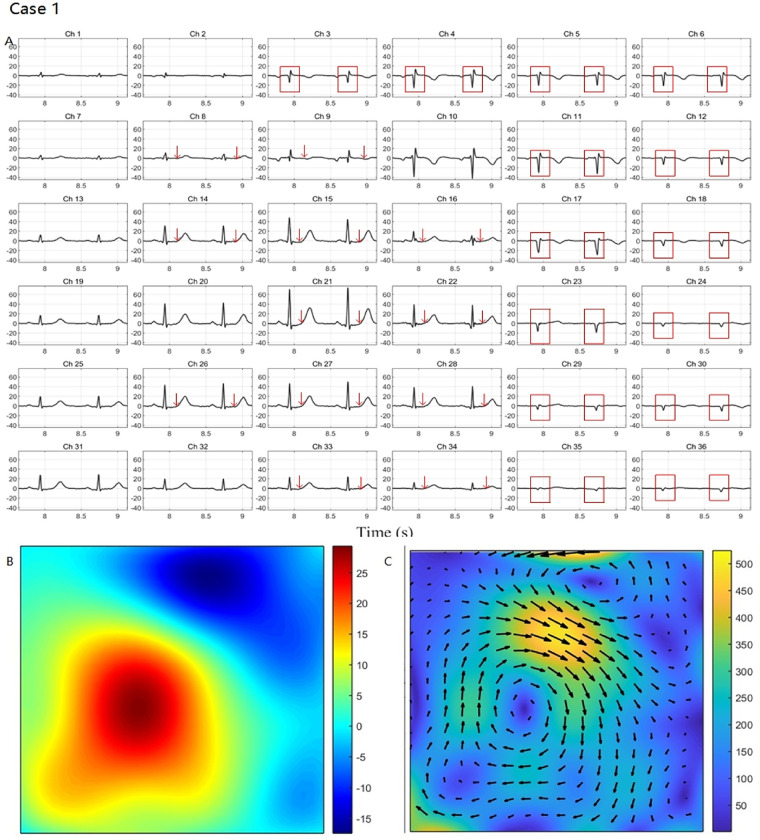
**(A)** MCG waveforms with ischemic features labeled; **(B)** *T*-wave peak magnetic field map; **(C)** *T*-wave peak pseudo current density map; **(D)** 12-lead ECG showing sinus rhythm without ischemic changes or arrhythmia; **(E)** coronary angiography demonstrating normal patent coronary arteries without stenosis, occlusion or plaque, including the left anterior descending, left circumflex, left main, and right coronary arteries in corresponding subpanels. **(F)** CMR sequences including contrast-enhanced imaging and LGE. Cine imaging revealed a dilated left ventricle with globally reduced systolic function. Focal wall thinning was noted in the apical portion of the interventricular septum. On first-pass perfusion, the left ventricular myocardium showed heterogeneous enhancement. LGE imaging demonstrated band-like hyperenhancement within the interventricular septum and inferior wall, with a focal transmural pattern in the basal to mid inferior wall.

Standard routine laboratory tests, including complete blood count and basic metabolic panel, were within normal ranges. A targeted D-dimer assessment was obtained concurrently with the first troponin measurement for the exclusion of pulmonary embolism, yielding a normal result (0.002 mg/L FEU, reference <0.5 mg/L FEU). Serial measurements of high-sensitive cardiac troponin I (hs-cTnI) were 1.91 ng/mL at presentation and 3.7 ng/mL at 1 h after admission, both significantly above the upper limit of normal (0.012 ng/mL). Following ECG examination, serum myocardial enzymes were assayed, and 36-channel SERF-MCG was performed 17 min later. The quantitative data for all MCG parameters are summarized in Table 3, with the complete dataset for Case 1 provided in the supplementary Excel file. Specifically, pathological *Q*-wave amplitudes were observed to exceed 10 times the upper limit of the normal reference range in channels 2, 5, 6, and 17, and reached more than 15 times the upper limit in channels 3, 4, 9, and 11 (Table 2). The ST segments also exhibited significant depression: the amplitudes at the onset (K1) decreased by more than 10-fold in channels 1, 2, 8, 17, 24, and 31; at the quarter interval (K2) in channels 6, 8, 19, 26, and 27; at the three-quarter interval (K3) in channels 6, 18, and 28; and at the endpoint (K4) in channels 6 and 17 (Supplementary Table 2). These quantitative findings were largely consistent with the results of the concurrent visual interpretation. The latter identified diffuse down-sloping ST-segment depression/flattening with *T*-wave flattening in channels 6, 9, 14–16, 21–22, 26–28, 33, and 34, as well as pathological *Q* waves in channels 3–6, 11–12, 17–18, 23, 24, 29, 30, 35, and 36 (Figure 3A). Meanwhile, abnormal magnetic field distribution and current density maps were observed at the positive poles at the *T*-wave peak (Figures 3B,C). Echocardiography revealed left ventricular dilatation, with a left ventricular ejection fraction (LVEF) of 45%. Given the elevated cTnI levels and positive findings on MCG, the patient underwent emergency coronary angiography (CAG) via the green channel approximately 2 h after presentation, which revealed no abnormalities. Additionally, quantitative flow ratio (QFR) was measured, with the following results: left anterior descending artery (LAD): 0.90; left circumflex artery (LCX): 0.99; right coronary artery (RCA): 0.96 (Figure 3E).

**Table 3:** *Q*_amp, *R*/*T* ratio, and *T*_amp for case 1 (36-channel MCG).

NO.	*Q*_amp (pT)	*R*/*T* Ratio	*T*_amp (pT)	NO.	*Q*_amp (pT)	*R*/*T* Ratio	*T*_amp (pT)	NO.	*Q*_amp (pT)	*R*/*T* Ratio	*T*_amp (pT)
1	1.556 ↑	1.906↓	2.631	2	5.077 ↑↑	160.527	0.03	3	15.924 ↑↑↑↑	2.52	4.568
4	24.934 ↑↑↑↑	1.5	9.673	5	20.012 ↑↑	0.946	8.264	6	20.012 ↑↑	0.907	8.203
7	1.65	1.482	3.09	8	2.202	1.641	4.906	9	9.997 ↑↑↑↑	10.634	1.702
10	0.438	1.841	12.647	11	30.432 ↑↑↑↑	1.067	10.78	12	15.375 ↑	0.574	5.568
13	2.163	2.01	6.087	14	3.66	2.059	15.103	15	3.049	2.086	21.994
16	2.254	1.985	8.51	17	25.364 ↑↑	0.916	5.535	18	15.018 ↑	0.443	3.862
19	2.221	2.253	7.377	20	4.105	2.332	17.861	21	5.737	2.464	28.95
22	11.876 ↑	2.61	15.176	23	19.385 ↑	0.596	2.529	24	6.301	0.633	1.55
25	2.603	2.416	8.13	26	4.363 ↑	2.618	16.818	27	4.237	2.804	16.925
28	4.155	2.924	13.518	29	8.332 ↑	2.207	1.306	30	8.585 ↑	0.542	2.954
31	3.259	2.56	10.794	32	2.816	2.582	7.444	33	3.132	2.987	7.763
34	2.816	2.975	4.095	35	3.803	5.701	0.275	36	5.507 ↑	2.348	0.72

↑, ↑↑, ↑↑↑, ↑↑↑↑ indicate values elevated to 1–5, 5–10, 10–15, and ＞15 times the upper confidence interval limit, respectively; ↓ indicates values reduced to 1–5 times the lower confidence interval limit.

**Figure:**
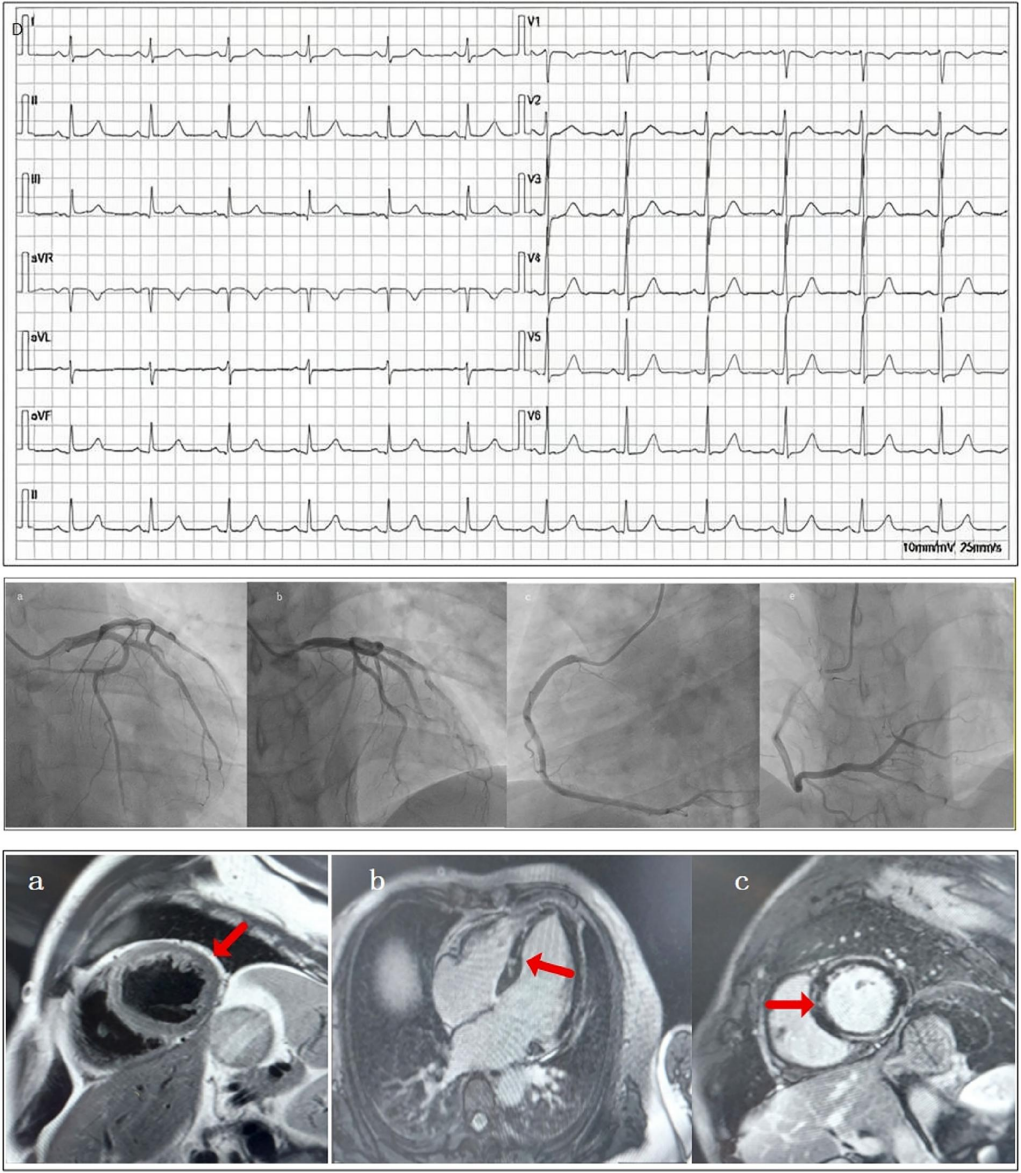


The patient's baseline antihypertensive therapy was continued throughout the initial diagnostic workup (including blood tests, ECG, MCG, and echocardiography), all of which were completed within 90 min of admission. Immediately after angiography, standard anti-ischemic therapy comprising aspirin, ticagrelor, rosuvastatin calcium, and nicorandil was initiated.

To confirm the diagnosis of MINOCA and explore its underlying pathophysiological mechanisms, the patient underwent CMR (Figure 3F). Cine imaging revealed a dilated left ventricle with globally reduced systolic function. Focal wall thinning was noted in the apical portion of the interventricular septum. On first-pass perfusion, the left ventricular myocardium showed heterogeneous enhancement. Late gadolinium enhancement (LGE) imaging demonstrated band-like hyperenhancement within the interventricular septum and inferior wall, with a focal transmural pattern in the basal to mid inferior wall. These CMR findings were consistent with the myocardial infarction pattern identified by MCG waveform analysis. Based on the comprehensive assessment, the patient was ultimately diagnosed with MINOCA.

### Case 2

The patient was a 45-year-old man who presented to the cardiology outpatient clinic with a chief complaint of chest pain. The chest pain was located in the sternal region, characterized by a squeezing and pressure-like sensation, and lasted approximately 2 h. He reported recurrent chest pain over the past month. On presentation, vital signs on room air were as follows: temperature 36.3 °C, heart rate 77 bpm, respiratory rate 18 bpm, and blood pressure 154/107 mmHg. Physical examination revealed no abnormal findings, and the patient denied a history of hypertension, diabetes mellitus, hyperlipidemia, alcohol consumption, or tobacco use. The patient had no prior medication history.

**Figure 4:**
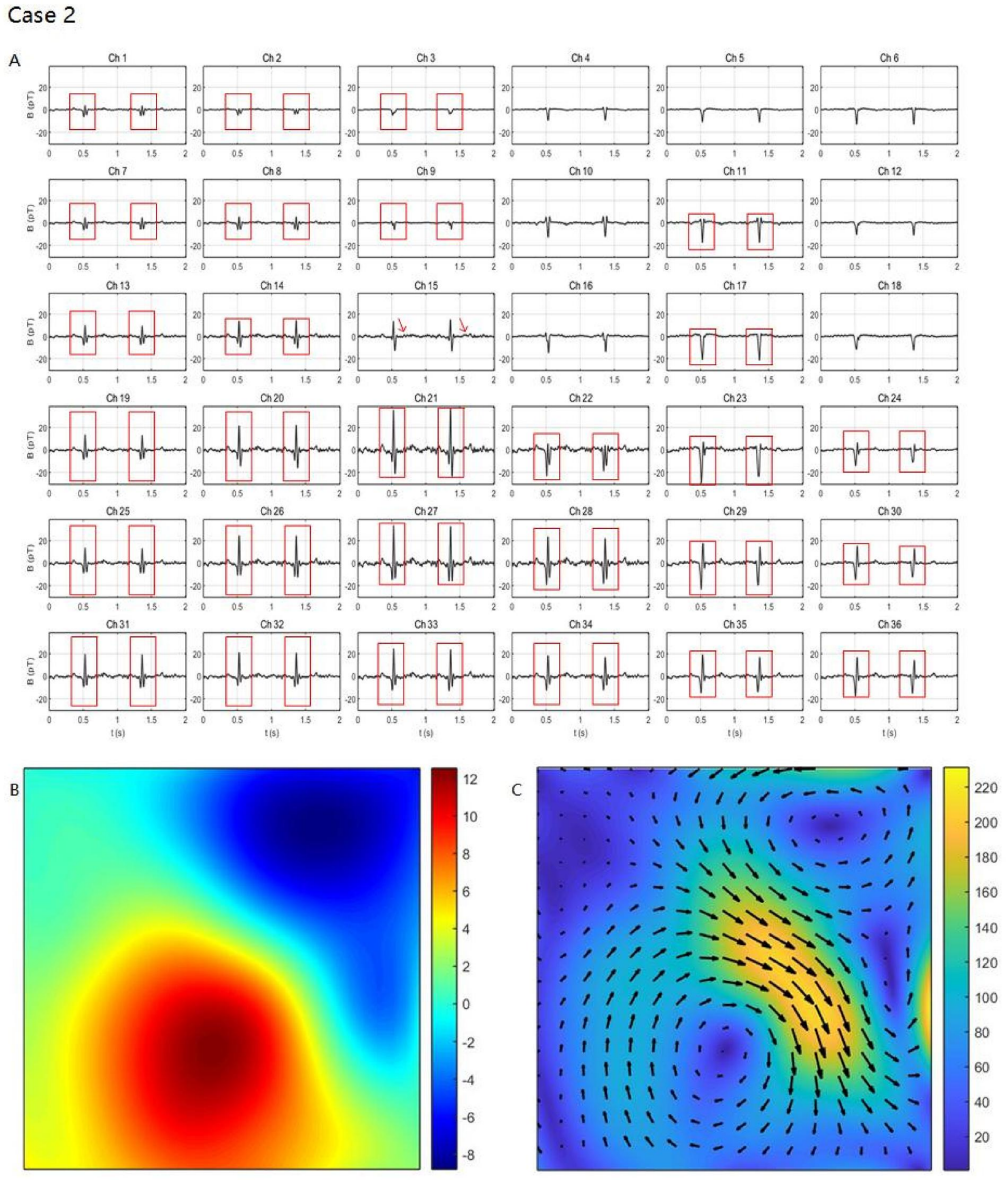
**(A)** labelled MCG waveforms showing ischemic features; **(B)** T-peak magnetic field map; **(C)** T-peak pseudo current density map; **(D)** 12-lead ECG showing sinus rhythm without ischemia or ectopy; **(E)** normal coronary angiography with no stenosis, occlusion or plaque, including the left anterior descending, left circumflex, left main, and right coronary arteries in corresponding subpanels; **(F)** CMR sequences including T1WI, T2WI, contrast-enhanced imaging and LGE. Patchy LGE hyperintensities were observed in the left ventricular lateral mid-wall and apical inferior wall, involving the subendocardium and partial mid-myocardium, with corresponding T2WI signal elevation; abnormal findings are marked by red arrows.

ECG showed nonspecific ST-T changes and sinus tachycardia, with a ventricular rate of 60 bpm (Figure 4D). A targeted D-dimer assay for differential diagnosis yielded a normal result of 0.0013 mg/L FEU (reference <0.5 mg/L FEU). The cardiac troponin I (cTnI) level was 0.81 ng/mL at presentation and increased to 1.05 ng/mL at 1 h after admission, both of which were significantly higher than the gender-specific normal cutoff of 0.023 ng/mL. Additionally, the creatine kinase-myocardial band (CK-MB) level was 64 ng/mL at baseline and rose to 78 ng/mL at 1 h (normal cutoff: 5 ng/mL), and the myoglobin level was 272 ng/mL on admission and increased to 310 ng/mL at 1 h (normal cutoff: 112 ng/mL). Echocardiography demonstrated mild tricuspid, mitral, and aortic valve regurgitation, along with left ventricular filling dysfunction. Eighteen minutes after ECG testing, he underwent 36-channel SERF-MCG. MCG analysis identified two primary anomalies. The quantitative data for all MCG parameters are presented in Table 4. Specifically, in channel 15, there was ST-segment depression/flattening and *T*-wave flattening, characterized by a low *T*-wave amplitude (1.895 pT; reference: 2.8169–3.1095 pT) and a high R/T ratio (11.498; reference: 8.493–9.361). Additionally, pathological *Q* waves with amplitudes >0 pT were automatically detected in 28 channels, with 5 and 10 channels exceeding the upper reference limit by >10-fold and 5–10-fold, respectively (Table 3). Manual review confirmed significant pathological *Q* waves in channels 11, 17, 23, 29, 30, and 36, showing good consistency with the automated findings (Figure 4A). Meanwhile, abnormal magnetic field distribution was observed at both the positive and negative magnetic poles at the *T*-wave peak, accompanied by current changes in the pseudo current density map at the same time point (Figures 4B,C).

**Figure:**
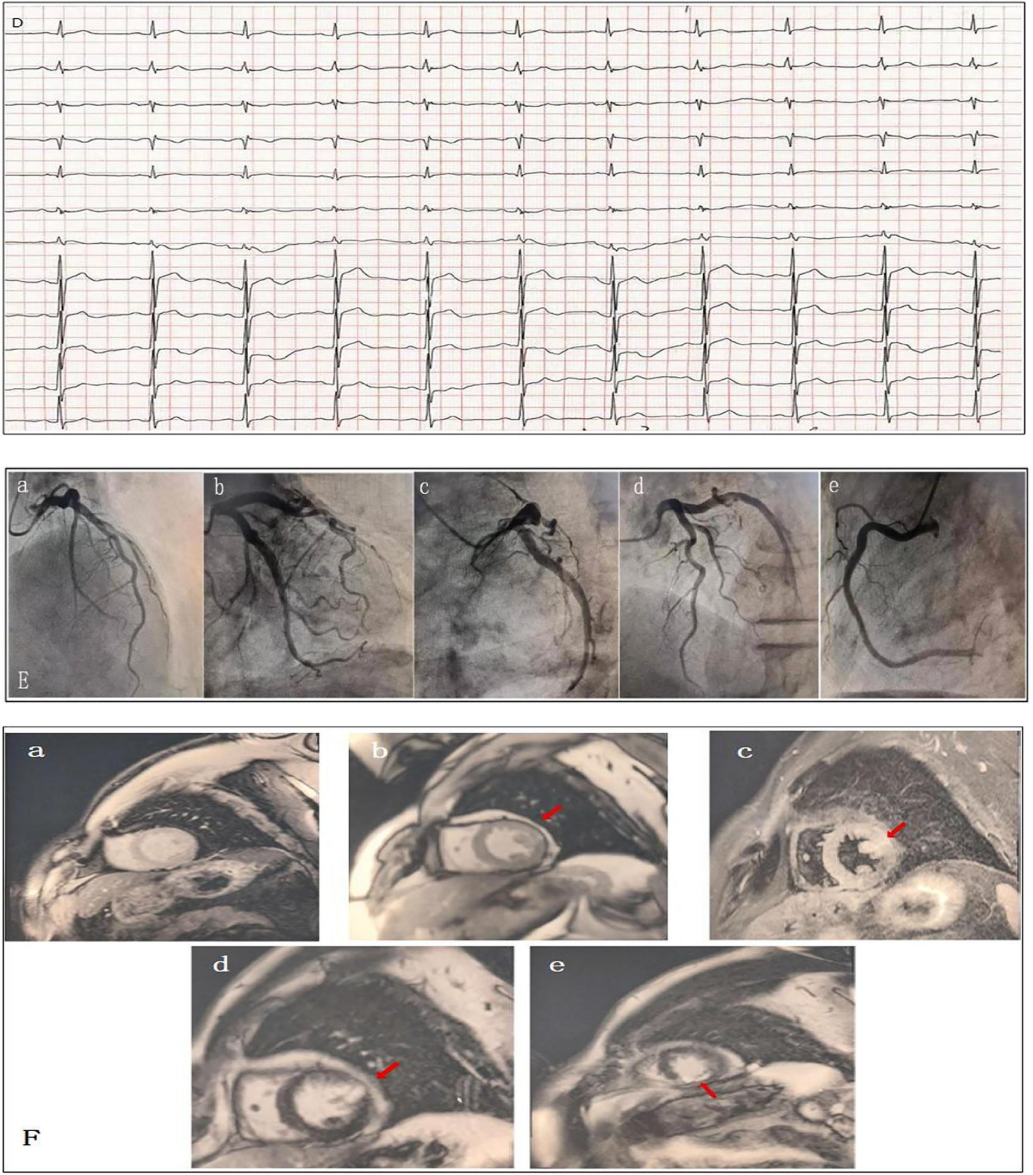


**Table 4:** *Q*_amp, *R*/*T* ratio, and *T*_amp for case 2 (36-channel MCG).

NO.	*Q*_amp (pT)	*R*/*T* Ratio	*T*_amp (pT)	NO.	*Q*_amp (pT)	*R*/*T* Ratio	*T*_amp (pT)	NO.	*Q*_amp (pT)	*R*/*T* Ratio	*T*_amp (pT)
1	6.116 ↑↑↑	2.126	1.073	2	5.161 ↑ ↑	4.467	0.653	3	5.572 ↑ ↑	2.701	1.778
4	10.488 ↑↑↑	1.951	2.748	5	15.609 ↑	1.911	2.673	6	16.659 ↑	1.834	2.459
7	6.106 ↑↑↑	4.534	0.584 ↓	8	5.022 ↑↑↑	6.443	0.593 ↓	9	7.805 ↑↑↑	10.373	0.728 ↓
10	0.148	2.451	4.331	11	0.663	1.768	4.011	12	10.694 ↑	1.893	2.21
13	6.324 ↑ ↑	4.112	1.534 ↓	14	5.301 ↑ ↑	5.807	3.049	15	0.014	11.498↑	1.895 ↓
16	1.199 ↑	90.987↑↑↑	0.094 ↓	17	20.564 ↑ ↑	2.509	3.049	18	11.092 ↑	2.794	1.119 ↓
19	6.084 ↑↑	8.52	1.245 ↓	20	4.112 ↑	4.889	5.436	21	4.723 ↑	5.204	7.889
22	22.062 ↑	8.308	4.621	23	26.234 ↑	20.105↑	0.39 ↓	24	10.702 ↑	16.776 ↑	0.445 ↓
25	6.05 ↑↑	4.642	2.602	26	11.609 ↑↑	4.68	5.139	27	17.843 ↑↑	4.798	7.957
28	20.163 ↑	5.065	3.93 ↓	29	21.218 ↑	5.489	2.243	30	10.829 ↑	25.698 ↑	0.57 ↓
31	6.052 ↑↑	4.931	2.968	32	5.788 ↑	4.823	3.21	33	11.523 ↑	5.118	4.517
34	11.013 ↑	5.428	3.349	35	16.16 ↑	5.664	1.742	36	18.601 ↑	5.902	1.459

↑, ↑↑, and ↑↑↑ indicate values elevated to 1–5, 5–10, and 10–15 times the upper confidence interval limit, respectively; ↓ indicates values reduced to 1–5 times the lower confidence interval limit.

After completion of the initial noninvasive workup, CAG was subsequently performed to evaluate for coronary artery stenosis, which revealed no significant epicardial coronary narrowing. Additionally, quantitative flow ratio (QFR) measurements yielded the following results: LAD: 0.97; LCX: 0.98; RCA: 0.99; LM: 0.99 (Figure 4E). Based on the patient's elevated cardiac enzymes, ECG without ST-segment elevation, and presence of myocardial ischemic symptoms, NSTEMI was suspected initially.

To confirm the diagnosis of MINOCA and explore its underlying pathophysiological mechanisms, the patient underwent CMR (Figure 4F). On LGE imaging (Panels d and e), patchy hyperintensities are noted in the mid-segment of the left ventricular lateral wall and apical segment of the inferior wall, involving the subendocardium and partial mid-myocardial layer. The lateral wall hyperintensity exhibits a focal transmural-like pattern, with corresponding signal elevation in the affected myocardial segments on T2WI (Panel b). These CMR findings were consistent with the myocardial infarction pattern identified by MCG waveform analysis.

Similarly, no medications were administered during the initial diagnostic workup, including ECG, MCG, cardiac biomarker testing, and echocardiography. Anti-ischemic medications were administered during coronary angiography and cardiac CMR (performed 1 day after admission) for symptom relief. Subsequent to the review of the angiographic and CMR findings, a standard anti-ischemic regimen including aspirin, clopidogrel, atorvastatin, and metoprolol succinate was immediately initiated for the prevention of myocardial ischemia and adverse cardiac events, despite the absence of hypertension and hyperlipidemia.

Based on the integrated clinical, laboratory, and multimodality imaging findings, a final diagnosis of MINOCA was established.

### Differential diagnosis

Both patients presented with acute chest pain and dynamic elevation of cardiac biomarkers, and underwent coronary angiography showing unobstructed coronary arteries. These findings, combined with confirmatory CMR results, supported the final diagnosis of MINOCA. Key differential diagnoses were systematically excluded:

Myocarditis represents the primary differential diagnosis for MINOCA, as it frequently presents with chest pain, troponin elevation, and unobstructed coronary arteries. This diagnosis was excluded in both cases: neither patient had a history of recent viral infection, fever, or prodromal symptoms suggestive of acute inflammatory myocardial injury. In Case 2, CMR demonstrated a focal, subendocardial-dominant LGE pattern pathognomonic for ischemic myocardial infarction, which is distinct from the typical epicardial, mid-wall, or diffuse LGE pattern of myocarditis. In Case 1, the absence of inflammatory symptoms and clinical course further ruled out this diagnosis.

Ascending aortic dissection (Stanford Type A) can present with anterior chest pain, secondary myocardial ischemia, and troponin elevation if coronary ostia are compromised. This diagnosis was ruled out in both patients by stable vital signs, absence of back pain, and normal echocardiographic findings excluding aortic root dilation, dissection flap, or pericardial effusion.

Acute pulmonary embolism (PE) may mimic acute myocardial infarction with chest pain, troponin elevation, and right ventricular strain. This diagnosis was excluded in both cases: D-dimer levels were within normal limits in both patients (Case 1: 0.002 mg/L FEU; Case 2: 0.0013 mg/L FEU; both <0.5 mg/L FEU), ruling out the possibility of significant venous thromboembolism. Additionally, both patients had normal respiratory rates, no dyspnea, and echocardiography showing no right ventricular dilation, pressure overload, or thrombus, which are hallmark findings of significant PE.

Takotsubo cardiomyopathy (stress cardiomyopathy) presents with chest pain, troponin elevation, and unobstructed coronary arteries, typically triggered by severe emotional or physical stress. This diagnosis was ruled out in both patients by the absence of a clear stress trigger, and imaging findings inconsistent with the characteristic apical ballooning pattern of the disease. In Case 2, CMR confirmed focal ischemic LGE, which is not consistent with stress cardiomyopathy.

Stress-induced myocardial injury was also considered as a potential differential diagnosis. Neither patient had a history of recent surgery, trauma, severe systemic illness, or extreme physical/emotional stress, making this diagnosis highly unlikely. The recurrent chest pain and dynamic biomarker elevation in both cases were consistent with acute ischemic injury rather than stress-related myocardial damage.

After systematically excluding the above alternative diagnoses, both cases received a definitive diagnosis of MINOCA confirmed by CMR.

## Discussion

The inconsistency between symptom severity, ECG findings, and troponin levels often introduces diagnostic uncertainty, necessitating additional imaging and functional testing to identify the underlying etiology (8). As reported by Isabella Leo et al., even with the adoption of an unconventional time cutoff, the proportion of MINOCA patients with an unclear diagnosis remains as high as 27%—a finding that underscores the persistence of this diagnostic challenge (9). Notably, the timing of diagnostic testing is critical in the acute phase of MINOCA. CMR—the guideline-endorsed gold standard for MINOCA diagnosis—may yield false-negative results if performed too late (i.e., beyond 7–12 days after symptom onset) (10–12). Such false negatives can lead to delayed or missed diagnosis, which in turn increases the risk of adverse cardiovascular events and may have fatal consequences for patients (13, 14). To date, emergency and cardiology physicians still rely on traditional triage tools—including clinical symptoms, medical history, 12-lead ECG, and troponin assays—that cannot reliably rule out evolving acute myocardial infarction in the early phase. Collectively, these challenges—including the limitations of traditional triage modalities and the risks of diagnostic delay/missed diagnosis due to suboptimal examination timing—have created an urgent unmet clinical need for more effective and accurate diagnostic approaches that streamline and accelerates the diagnostic workflow. It is this unmet need that has spurred the exploration of novel diagnostic technologies such as MCG.

Unlike CCTA, whose specificity drops from ∼79% to ∼17% in high-CACS patients, MCG retains stable sensitivity (∼73%–76%) and specificity (∼83%–93%), outperforming CCTA for ischemia detection in calcified patients (15). Furthermore, MCG also surpasses conventional 12-lead ECG. High-dose dobutamine stress testing confirmed that MCG achieves markedly higher sensitivity (97.6% vs. 26.2%) for detecting significant coronary stenosis, with both methods showing equivalent specificity (82.8%) (16). Importantly, our team further verified this advantage in a large cohort of 4,500 MCG recordings: among patients with epicardial coronary stenosis, the PPV of MCG reached 0.90, compared with only 0.50 for conventional ECG under identical clinical conditions.

MCG's core advantage over conventional ECG lies in its far higher sensitivity for detecting early, ECG-undetectable myocardial ischemia, driven by its comprehensive assessment of cardiac electrophysiology (17). Recent advancements in SERF technology have enabled unshielded MCG operation, making it clinically feasible in routine hospital settings, with our in-house system compatible with catheterization laboratory environments (18–23). This unique capability stems fundamentally from the distinct physical principles and operational mechanisms that distinguish SERF-MCG from conventional SQUID-based systems. SQUID magnetometers are based on the superconducting Josephson effect, in which extremely weak magnetic fields are measured by detecting changes in magnetic flux in a superconducting loop, and operation under cryogenic conditions is required; in contrast, SERF magnetometers are based on the spin polarization and Larmor precession of alkali-metal atoms, where spin-exchange relaxation is suppressed under near-zero magnetic field and high atomic density conditions, and magnetic field information is read out optically at near-room temperature, with the fundamental difference being that the former relies on superconducting quantum interference to measure magnetic flux, whereas the latter relies on atomic spin quantum states to sense magnetic fields.

Nevertheless, engineering advancements alone are insufficient to facilitate the widespread clinical adoption of MCG. A major lingering challenge lies in MCG data interpretation, which continues to perplex clinicians: the lack of immediate, clear-cut interpretation criteria and uncertain diagnostic applicability represent the current biggest dilemmas. Beyond artificial intelligence (AI)-driven intelligent assessment models, the most urgent need for MCG today is the establishment of a standardized operational framework in medical centers—similar to that of ECG—based on subjective visual interpretation. Unfortunately, existing two-dimensional MCG representations (including magnetic field maps and current density maps) have poor interpretability. Disease-related abnormal changes in these maps manifest as a uniform pattern: specifically, “dispersed or split organized magnetic dipole orientation during the repolarization phase.” (24, 25) This lack of specificity underscores their inadequacy in meeting clinical interpretation needs. Therefore, we revisited the one-dimensional waveform first described by Baule in 1962 as a potential solution (26).

Both cases in this study underscore the clinical significance of rapid, accurate, and subjectively visually interpretable diagnostic tools for patients with MINOCA. When existing non-invasive modalities fail to provide accurate information for diagnosing or ruling out suspected myocardial infarction with non-obstructive coronary, MCG emerges as a viable alternative. Several studies have explored the diagnostic value of MCG for NSTEMI. For instance, Lim et al. analyzed four types of MCG maps in normal controls vs. NSTEMI patients and found that NSTEMI patients exhibited more abnormal MCG parameters (27). Unlike previous studies, however, we are the first to perform one-dimensional MCG waveform analysis in patients with MINOCA—a less severe subtype of NSTEMI compared with obstructive NSTEMI. As illustrated in Figure 2, we propose that, similar to the ECG, the presence of abnormal pathological *Q* waves represents a specific feature of various types of MI, whereas ST-T segment changes merely indicate myocardial ischemia. Notably, pathological *Q* waves identified on MCG differ from those on ECG; MCG-defined pathological *Q* waves include deep-amplitude *Q* waves, *Q*r waves, fragmented *Q* waves, and qRs waves appearing in channels where *Q* waves are not typically expected (e.g., channels 25–27, 31–34; Figure 3). This distinction is mechanistically plausible: during myocardial ischemia, biological injury currents and repolarization abnormalities induce abnormal MCG morphologies during the ST interval and T wave (28). In contrast, following myocardial necrosis, the loss of electrical activity in the local myocardium disrupts the normal myocardial depolarization sequence and electrical vector conduction, ultimately manifesting as changes in the morphology, duration, or voltage of the QRS complex (29, 30).

Driven by technological advances in sensor design and noise reduction—especially the development of SERF-MCG—MCG has evolved from a research tool to a clinically viable modality (31, 32). The gold-standard cryogenic SQUID-based systems are now complemented by compact, cost-effective non-cryogenic alternatives (OPMs and SERF atomic magnetometers) that obviate helium cooling (33). Coupled with active shielding and AI-driven denoising, unshielded MCG operation is now feasible in standard hospital settings, with acquisition and automated analysis shortened to under five minutes for high-throughput clinical workflows. Regulatory approval (e.g., Health Canada's CardioFlux MCG approval for myocardial ischemia diagnosis) and large-scale trials [MAGNETO (34), MICRO (35)] have validated its translational value. MCG detects ECG-undetectable “electrically silent” repolarization abnormalities and circular currents, enabling early myocardial ischemia identification and arrhythmic risk stratification in populations with inconclusive ECG results, including acute chest pain, MINOCA, and CMVD (36, 37).

It is proposed that ischemic MINOCA can be diagnosed when the following conditions are met: patients present with elevated serum myocardial enzymes, accompanied by abnormal *Q* waves and flattened ST-T segments on MCG—rather than other abnormal changes such as ST-segment elevation (coved type) or high QRS wave amplitude. Meanwhile, coronary angiography or CTA confirms the absence of coronary artery stenosis. In such cases, there is no need for subsequent cardiac magnetic resonance (CMR) examination for confirmation, nor is ECG necessary. This diagnostic approach leverages the characteristic MCG manifestations to optimize the diagnostic pathway for MINOCA, reducing unnecessary supplementary examinations while ensuring diagnostic accuracy.

Beyond its utility for the initial screening of MINOCA, we further propose integrating spasm provocation testing and investigations of coronary vasomotor disorders into a non-invasive preliminary diagnostic protocol. This integration aims to gain deeper insights into the potential pathophysiological mechanisms of MINOCA, given the significant heterogeneity in its underlying etiologies. In practical practice, non-invasive assessment of coronary spasm and coronary vasomotor dysfunction can be achieved by comparing MCG images of patients before and after administration of acetylcholine or adenosine. This approach allows for the identification or exclusion of epicardial coronary artery spasm and coronary microvascular dysfunction. The core logic of this diagnostic model—observing indicator changes following pharmacological intervention to assist in etiological identification—is highly consistent with the clinical practice of performing the propranolol test using ECG. Such etiological clarification, in turn, facilitates the implementation of personalized management strategies for MINOCA, which should be tailored to the underlying cause of the patient's condition.

Nevertheless, this study has several limitations that need to be addressed. First, the presented data come only from case reports, and therefore require verification in clinical trials, which would allow for statistical analysis of data collected on a larger number of patients and determine the predictive value of MCG in the diagnosis of MINOCA. Second, the ECG-like MCG waveform interpretation method used in this study is a novel approach first proposed by our team. While the core diagnostic features have been preliminarily validated in our previous large-scale machine learning cohort (*n* = 4,500 MCG recordings, under review), unified standardized diagnostic thresholds and systematic interobserver variability data have not yet been fully established in independent prospective cohorts, which requires further investigation in future studies. We acknowledge that the credibility of this newly proposed diagnostic standard may be questioned; accordingly, we note that our criteria are consistent with well-validated ECG principles for myocardial ischemia/infarction, and preliminary validation in our large cohort provides initial support for its reliability. Future prospective studies will further verify and standardize this framework to enhance its clinical credibility. Third, we did not collect MCG data from MINOCA mimickers to analyze their waveform features and further exclude these confounding conditions. Finally, we did not investigate the MCG characteristics of spontaneous coronary artery dissection—a key subtype of MINOCA—which limits our ability to distinguish between MINOCA subgroups with different pathological mechanisms for differential diagnosis. This is particularly notable because accurate differentiation of underlying etiologies is critical for guiding subsequent investigations and clinical management.

## Conclusion

This case series highlights that our domestically developed high-performance SERF-MCG systems provide a valuable diagnostic approach for the MINOCA scenario, and our proposed interpretation criteria for myocardial infarction-related waveform patterns may facilitate the wider clinical adoption of this modality. These cases demonstrate that 180-second MCG can reliably diagnose MINOCA in complex clinical scenarios, and that MCG and CMR—with the former providing functional imaging of cardiac electrophysiological activity and the latter offering detailed anatomical and tissue characterization—serve as highly complementary modalities. Given that current international guidelines prioritize non-invasive cardiac imaging techniques in the management of acute coronary syndrome patients with non-obstructive coronary arteries (including MINOCA), MCG may be fully considered for integration into this management pathway. This is particularly relevant for the subset of MINOCA patients who present with normal ECG or cardiac troponin levels on admission.

Looking forward, the diagnosis of MINOCA—especially in patients with normal initial ECG or cardiac troponin—remains an area with unmet clinical needs. This novel method (MCG) offers unique value for these patients by effectively filling this diagnostic gap, in stark contrast to conventional ECG, which fails to address such unmet needs.

## Data Availability

The original contributions presented in the study are included in the article/Supplementary Material, further inquiries can be directed to the corresponding authors.

## Appendix

### Establishment of Normal Reference Ranges​

To define the normal reference ranges for MCG waveform parameters, we conducted a retrospective analysis of 1,011 adult subjects from a multicenter health screening database (Qilu Hospital of Shandong University, Linyi Hospital, and Guangdong Provincial Hospital of Chinese Medicine) collected between May 2021 and December 2024. Inclusion criteria included age 18-80 years and absence of self-reported cardiovascular disease, hypertension, or diabetes, as well as no related clinical symptoms. Exclusion criteria were applied for individuals with abnormal ECG findings, echocardiographic evidence of structural or functional heart disease, or prior cardiac interventions. All participants underwent standardized diagnostic evaluation to confirm eligibility. The final reference intervals were calculated as the 2.5th and 97.5th percentiles of the parameter distributions across the entire healthy cohort, ensuring robust representation of physiological variability. Waveform parameters were automatically analyzed, including T_amp, ratio_RT, and four amplitude points (k1amp–k4amp) obtained by equally dividing the ST segment from onset to offset into three segments.

**Table 1.** Normal Reference Ranges for MCG Waveform Parameters (Central 95% Interval)

	Definition	Feature	Reference
ST segment	Four amplitude points (k1–k4) obtained by equally dividing the ST segment from onset to offset into three segments.	k1amp	[0.114pT, 0.128pT]
		K2amp	[0.391pT, 0.421pT]
		K3amp	[0.745pT, 0.800pT],
		K4amp	[0.754pT, 0.819pT]
		ST-segment Morphology	Mild upward-sloping ST elevation, with no significant horizontal or downsloping ST depression.
T waveform	1) T amplitude: maximum magnetic field strength from isoelectric baseline to T-wave peak 2) R/T ratio: ratio of R-wave amplitude to T-wave amplitude	T amplitude	[2.858pT, 2.917pT]
		R/T ratio	[4.802, 4.891]
		T-Wave Morphology	In the R-wave positive channel, the T-wave is upright, without biphasic or inverted morphology.
