# Supplementary material for: Magnetocardiography in diagnosis of MINOCA: a case series
Source: Front Cardiovasc Med. 2026 Jul 15;13:1759132. doi: 10.3389/fcvm.2026.1759132 (PMC13416843; doi:10.3389/fcvm.2026.1759132)
Supplement: Supplementary file 1 [file Supplementaryfile1.docx]

**Supplementary Materials**


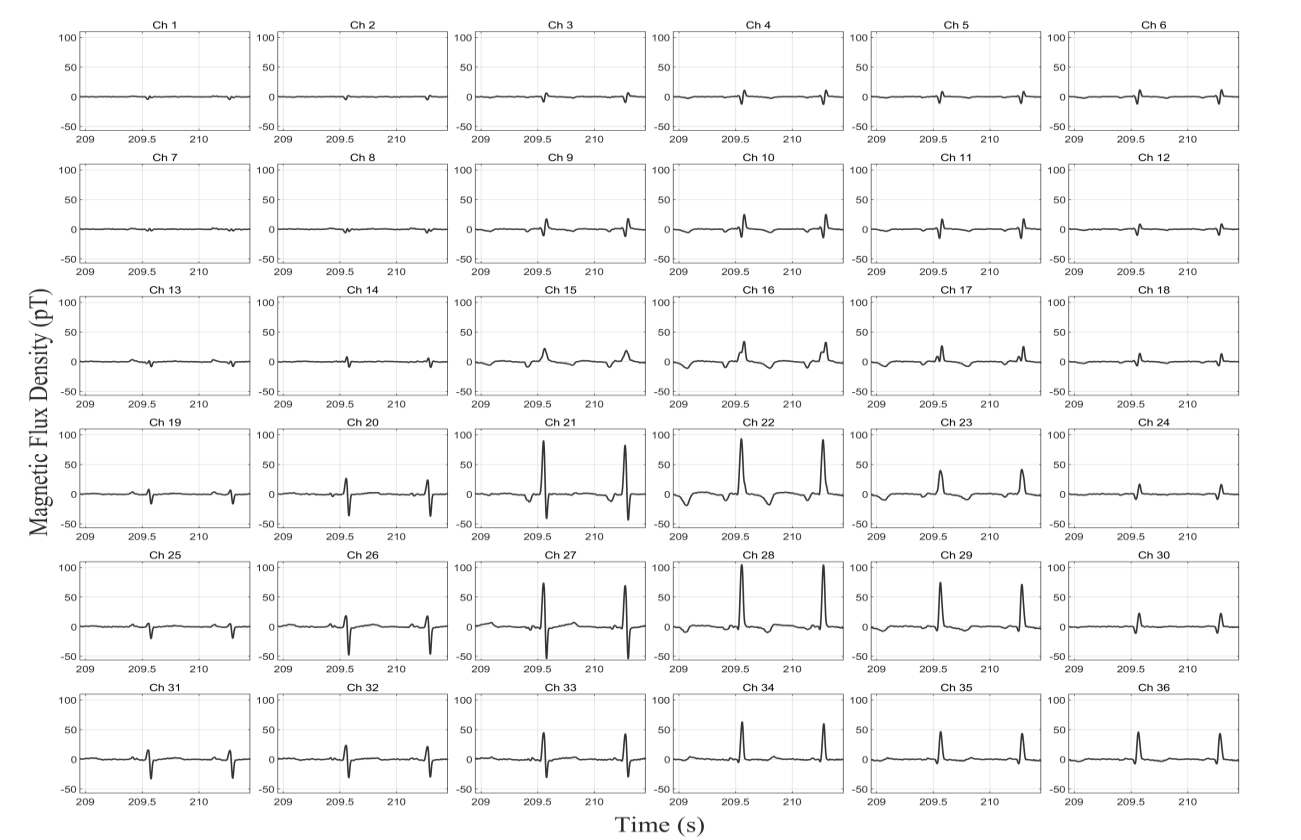


Figure 1. Representative MCG waveform recorded from a patient with dilated cardiomyopathy. Similar to the post-myocarditis case, this tracing also demonstrates the absence of pathological Q waves.

**Establishment of Normal Reference Ranges​**

To define the normal reference ranges for MCG waveform parameters, we conducted a retrospective analysis of 1,011 adult subjects from a multicenter health screening database (Qilu Hospital of Shandong University, Linyi Hospital, and Guangdong Provincial Hospital of Chinese Medicine) collected between May 2021 and December 2024. Inclusion criteria included age 18–80 years and absence of self-reported cardiovascular disease, hypertension, or diabetes, as well as no related clinical symptoms. Exclusion criteria were applied for individuals with abnormal ECG findings, echocardiographic evidence of structural or functional heart disease, or prior cardiac interventions. All participants underwent standardized diagnostic evaluation to confirm eligibility. The final reference intervals were calculated as the 2.5th and 97.5th percentiles of the parameter distributions across the entire healthy cohort, ensuring robust representation of physiological variability. Waveform parameters were automatically analyzed, including R_amp, T_amp, ratio_RT, Q_amp, and four amplitude points (k1amp–k4amp) obtained by equally dividing the ST segment from onset to offset into three segments. The complete statistical values (2.5th–97.5th percentiles, mean, standard deviation, median, and range) for all 36 MCG channels are provided in the supplementary Excel file attached to this article.

Table 1. Normal Reference Ranges for MCG Waveform Parameters (Central 95% Interval)

|  | Definition | Feature | Reference |
| --- | --- | --- | --- |
| ST segment | Four amplitude points (k1–k4) obtained by equally dividing the ST segment from onset to offset into three segments. | k1amp | [0.114pT, 0.128pT] |
|  |  | K2amp | [0.391pT, 0.421pT] |
|  |  | K3amp | [0.745pT, 0.800pT], |
|  |  | K4amp | \| [0.754pT, 0.819pT] \| \| --- \| |
|  |  | ST-segment  Morphology | Mild upward-sloping ST elevation, with no significant horizontal or downsloping ST depression. |
| T waveform | 1) T amplitude: maximum magnetic field strength from isoelectric baseline to T-wave peak  2) R/T ratio: ratio of R-wave amplitude to T-wave amplitude | T amplitude | [2.858pT, 2.917pT] |
|  |  | R/T ratio | [4.802, 4.891] |
|  |  | T-Wave Morphology | In the R-wave positive channel, the T-wave is upright, without biphasic or inverted morphology. |
| Pathological Q wave | Q amplitude: maximum negative magnetic deflection of the Q wave in MCG | Q waves exist, Q amp | channels 5, 6, 12, 17, 18, 21-24, 27-30, and 35-36 |
|  |  |  | [2.241pT, 2.302pT] |
|  |  | No Q waves exist | other channels. |

Table 2 K1–K4 Amplitude Values for Case 1 Across 36 MCG Channels

| NO. | K1amp (pT) | K2amp (pT) | K3amp(pT) | K4amp (pT) | NO. | K1amp (pT) | K2amp (pT) | K3amp(pT) | K4amp (pT) |
| --- | --- | --- | --- | --- | --- | --- | --- | --- | --- |
| 1 | -0.176 ↓↓↓ | -0.418↓↓↓↓ | -0.177↓↓ | -0.238 | 2 | -0.493↓↓↓ | -0.29 | 0.373 | 0.315 ↓↓ |
| 3 | 0.299 | 0.267↓↓ | 0.255 | 0.528↓ | 4 | 0.652 | 0.578↓↓ | 0.57 | 0.868↓ |
| 5 | 0.517 | 0.749↓↓ | 0.718 | 0.493↓ | 6 | -0.313 ↓ | -2.471↓↓↓↓ | -5.889↓↓↓ | -8.132↓ |
| 7 | -0.373 | -0.365 | -0.661 | -0.559↓↓↓↓ | 8 | -0.508 ↓↓ | -0.723 ↓↓↓ | -0.553 | -0.336↓↓↓↓ |
| 9 | 0.529 | 0.731↓↓ | 0.91 | 0.867 ↓↓ | 10 | 0.797 | 0.744↓ | 1.219 | 0.942 ↓↓ |
| 11 | -0.351 ↓ | -3.152↓↓ | -7.77↓ | -10.677↓ | 12 | 0.626 | 0.643↓↓ | 0.614 | 0.559↓ |
| 13 | -0.871 ↓↓ | -1.123 | -1.047↓ | -0.809↓ | 14 | 0.809 | 4.437 | 10.491 | 14.919↓ |
| 15 | 2.366 ↓↓↓↓ | 8.365↑ | 16.128 | 21.754 ↓↓ | 16 | -1.45 ↓↓↓ | -1.441 | -0.905↓ | -0.495↓↓↓↓ |
| 17 | -0.862 ↓ | -2.678↓↓ | -4.45↓ | -5.486↓↓↓↓ | 18 | -0.549 | -1.702↓↓ | -3.033↓↓ | -3.839 ↓↓ |
| 19 | 0.802 | 2.821↓↓↓↓ | 5.482 | 7.269↓↓↓↓ | 20 | 2.458 | 7.284 | 13.52 | 17.628↓↓↓↓ |
| 21 | 3.867 | 11.697 | 21.67 | 28.615↓ | 22 | 1.083 | 4.378 | 10.546 | 14.994↓ |
| 23 | -0.371 | -0.294 | 0.26 | 0.291↓↓↓ | 24 | -0.703 ↓↓ | -1.399 | -1.168 | -0.055 ↓↓ |
| 25 | 1.07 ↓↓ | 3.501 | 6.184 | 8.001↓ | 26 | 2.408 | 7.528↓↓↓↓ | 13.073 | 16.609↓↓↓ |
| 27 | 2.762 ↓ | 7.693↓↓↓↓ | 13.092 | 16.699↓ | 28 | 2.017 | 5.691 | 10.459↓↓↓↓ | 13.345↓ |
| 29 | -0.488 | -0.488 | -0.46 | -0.403↓ | 30 | -1.319 ↓↓ | -2.787↓↓ | -2.348 | 0.036↓ |
| 31 | 1.73 ↓↓↓↓ | 4.915 | 8.514 | 10.682↓ | 32 | 1.151 | 3.456 | 5.81↓ | 7.364↓↓↓ |
| 33 | 2.011 | 4.469 | 6.417 | 7.67 ↓↓ | 34 | -1.619 ↓↓↓↓ | -1.601 | -1.545 | -1.446↓ |
| 35 | -0.714↓↓ | -0.547 | -0.173 | -0.274↓ | 36 | -0.686 ↓↓↓↓ | -0.707↓ | -0.61 | -0.497↓ |

↓ , ↓↓, ↓↓↓, ↓↓↓↓ indicate values reduced to 1–5, 5–10, 10–15, and ＞15 times the upper confidence interval limit, respectively.
